# An unusual case of hypothermia associated with therapeutic doses of olanzapine: a case report

**DOI:** 10.1186/1752-1947-5-189

**Published:** 2011-05-18

**Authors:** Lalith RMR Rasnayake, Harith Wimalarathne, Runkman KTDP Jayapala, Chaminda DM Gamage, Dinesh LB Dassanayake, Shiroma L Ratnayake, Anuradha Colombage, Damith Nandadeva, Amitha NPK Nelumdeniya

**Affiliations:** 1 Medical Unit, Teaching Hospital, Kandy, Sri Lanka

## Abstract

**Introduction:**

We report a case of a 42-year-old man who had symptomatic hypothermia as a result of taking olanzapine for paranoid schizophrenia. According to published data, only a few cases of hypothermia associated with olanzapine have been reported since its introduction into clinical use.

**Case presentation:**

A 42-year-old Sri Lankan man with schizophrenia who was being treated with a therapeutic dose of olanzapine presented with reduced level of consciousness. He had a core temperature of 32°C and was bradycardic. At the time of admission, the electrocardiogram showed sinus bradycardia with J waves. He did not have any risk factors for developing hypothermia except the use of olanzapine. There was improvement in his clinical condition with reversal of electrocardiogram changes following gradual rewarming and the omission of olanzapine.

**Conclusion:**

Hypothermia induced by antipsychotic medications is not uncommon, but olanzapine-induced hypothermia is rare and occurrence has been reported during initiation or increasing the dose. But here the patient developed hypothermia without dose adjustment.

## Introduction

In homeothermic animals such as humans, body temperature is maintained within a narrow range by the hypothalamus. Hypothermia is traditionally defined as a drop in core body temperature below 35°C (95°F).

In clinical practice, the most common causes of hypothermia are prolonged exposure to cold temperature as well as extremities of age, malnutrition, hypoglycemia, adrenal insufficiency, hypothyroidism, diabetes mellitus, stroke, disability, sepsis, shock, burns and exfoliative dermatitis. After initial stimulation by hypothermia, there is progressive depression of all organ systems. Depending on the severity of the hypothermia, patients may show various clinical manifestations from shivering and a feeling of coldness to deep coma.

According to published data, only a few cases of hypothermia associated with olanzapine have been reported since its introduction into clinical use [1-5]. Temperature dysregulation is a known side effect of antipsychotic medication through their effects on hypothalamic neurotransmission. Recognized mechanisms include antagonism of the dopamine D_2 _[6] and 5-hydroxytryptamine _2 _(5HT_2_) receptors [7-9]. Neurotensin (NT) has also been recognized as a mediator of hypothermia, especially in patients with schizophrenia [10]. We report a similar case of hypothermia associated with olanzapine use.

## Case presentation

Our patient was a 42-year-old Sri Lankan man from a mountainous region close to Kandy. The usual environmental temperature of the patient's hometown is around 30°C. He was unmarried and lived with his parents. He was diagnosed with paranoid schizophrenia for the past six years and was prescribed olanzapine 10 mg daily since then. He did not have a history of diabetes mellitus, hypothyroidism, stroke, ischemic heart disease, adrenal insufficiency, hypopituitarism or arthritis predisposing him to hypothermia.

**Figure 1:**
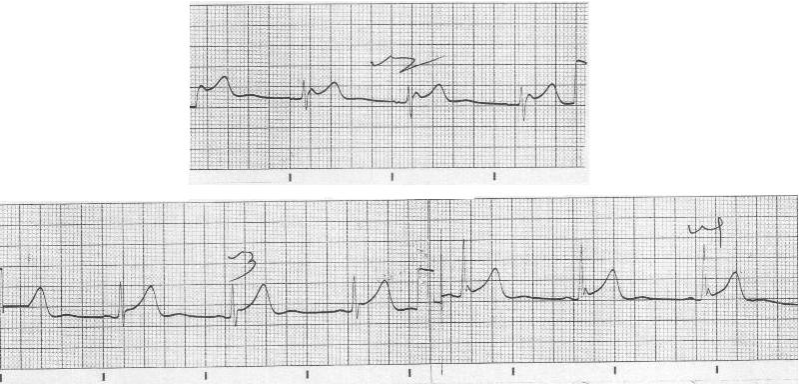
**Electrocardiogram (ECG) obtained at the time of admission showing sinus bradycardia with J waves **.

He was admitted to our hospital because he was found unresponsive in his room. He had been well the previous night. There was no history of substance ingestion, head trauma or drug overdose. At presentation, his Glasgow Coma Scale (GCS) score was 8/15. He opened his eyes to pain and had no verbal response, but was able to localize pain. His pulse rate was 48 beats/minute and regular and was low in volume. His blood pressure was 90/60 mmHg. His muscle tone was reduced, and reflexes in all four limbs were diminished with flexor plantar response. His oral temperature was 32°C. At the time of admission, his electrocardiogram (ECG) showed sinus bradycardia with J waves (Figure 1).

His random venous plasma sugar was 130 mg/dL, thyroid-stimulating hormone level was 2.2 mu/L and free thyroxin level was 2.8 u/dL. The patient's blood urea nitrogen was 5.5 mmol/L, Na was 135 mmol/L, K was 4.4 mmol/L and complete blood count was normal. His chest X-ray and contrast computed tomography of the head were also unremarkable.

On the basis of the clinical findings, the patient's body temperature and ECG findings, the diagnosis of hypothermia was made. The patient did not have any risk factors for developing hypothermia except for the use of olanzapine.

**Figure 2:**
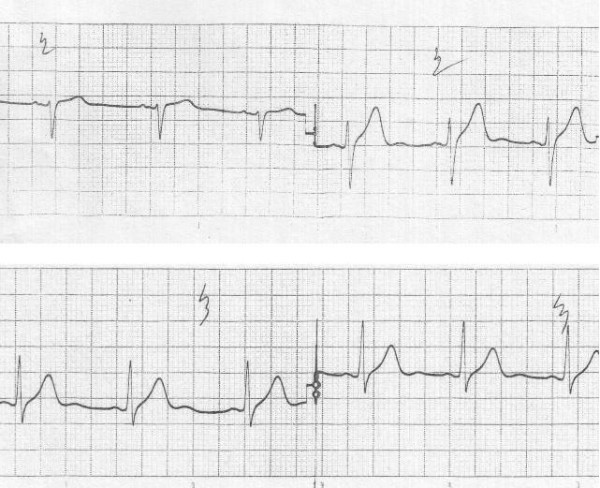
**ECG obtained after clinical improvement showing reversal of previous changes **.

He was rewarmed gradually with blankets and warm saline infusion. Olanzapine was discontinued. His level of consciousness, vital signs and cardiac rhythm were monitored. He gradually regained consciousness after 24 hours. After clinical improvement, the patient's body temperature was 38°C and the ECG changes had reversed (Figure 2).

## Discussion

Hypothermia in patients using antipsychotic drugs (APD) is a serious, unpredictable, type B adverse drug reaction and/or idiosyncratic reaction that frequently leads to hospital and intensive care unit admission and sometimes even to death.

According to published data in the World Health Organization database, atypical antipsychotic medications are the commonest antipsychotics which cause hypothermia. The commonest culprit is risperidone, and the most common psychiatric disorder associated with hypothermia is schizophrenia [11].

Antipsychotic medication-induced hypothermia could be mediated through its effects on antagonism of D_2 _receptors [6] and may be due to antagonism of the 5-HT_2 _receptors [7-9]. Olanzapine is an atypical antipsychotic which acts on both these receptors. Olanzapine is structurally similar to clozapine, but is classified as a thienobenzodizepine. Olanzapine has a higher affinity for 5-HT_2 _serotonin receptors than D_2 _dopamine receptors [11].

Like most atypical antipsychotics, compared to the older typical ones, olanzapine has a lower affinity for histamine, cholinergic muscarinic and α-adrenergic receptors.

Blocking α_2_-adrenergic receptors (for example, chloropromazine, risperidone, clozapine, thioridazine) may also increase the hypothermic effect by inhibiting peripheral responses to cooling (that is, vasoconstriction and shivering) [11].

In addition to these mechanisms, NT has been recognized as a mediator of hypothermia. In schizophrenia, thermal regulation is altered. This may be explained by changes in NT levels. NT is one of the most important thermoregulatory peptides that also play a role in the antipsychotic actions of APDs. In patients with schizophrenia, NT concentration in the cerebrospinal fluid (CSF) is low and is usually normalized following antipsychotic drug use [10]. The hypothermic reaction is also dependent on ambient temperature. In animal studies, APD administration at ambient temperatures below 22°C led to hypothermia, whereas APD administration in a room temperature of 29°C gave no thermal response and at 32°C led to an increase in rectal temperature [10,12]. Normally, a cold environment results in behavior aimed at protection against the cold (for example, taking extra blankets or clothes). APDs, however, induce apathy and indifference, resulting in unawareness of developing hypothermia.

There are only few reported cases of olanzapine-induced hypothermia. In general, an antipsychotic medication can be the sole cause of hypothermia, or it can be one of a number of possible causes coexisting in the individual patient who develops hypothermia. In one case, an older adult woman with schizophrenia but no other medical illness developed hypothermia (31.7°C) four hours after taking a single 10-mg dose of olanzapine [1]. She did not have any other medical illness predisposing her to hypothermia and recovered after discontinuation of olanzapine.

In another case, an 83-year-old woman with bipolar affective disorder developed hypothermia of 33.1°C after initiation of olanzapine 5 mg daily, but she was taking lithium carbonate, clonazepam and trazodone as well. Her body temperature was restored after cessation of all medication [2].

In a third case, a 37-year-old woman with Prader-Willi syndrome and psychotic disorder developed hypothermia on two occasions while being treated with risperidone and once following a single dose of olanzapine [3]. In this case, there were endocrine abnormalities as well as infections that may have contributed to the development of hypothermia. However, the patient had had previous episodes of infections while she was not taking antipsychotic medications and had not developed hypothermia during those episodes.

In the fourth case, a 64-year-old woman with a 40-year history of bipolar affective disorder developed hypothermia while on olanzapine, but she had subclinical hypothyroidism and type 2 diabetes mellitus, and in addition she was taking gabapentin [4]. These factors are known to cause hypothermia. However, she improved once olanzapine was discontinued and after levothyroxine therapy was initiated.

In the fifth case, an adolescent with schizophreniform disorder developed hypothermia and rhabdomyolysis simultaneously after intramuscular injection of olanzapine. A 17-year-old boy was hospitalized for treatment of psychotic symptoms, which persisted on risperidone 3 mg/day for three weeks. Then his antipsychotic drug was shifted to oral olanzapine 10 mg/day. The next day he received an intramuscular injection of olanzapine 5 mg and soon developed hypothermia, rhabdomyolysis, hypotension and bradycardia. These symptoms subsided gradually in the next two weeks after supportive treatment was given [5].

In our patient, the general medical conditions predisposing them to the development of hypothermia, such as hypoglycemia, adrenal insufficiency, diabetes mellitus, shock, burns, exfoliative dermatitis, hypothyroidsm, malnutrition and sepsis were not found. But it is shown in the literature that schizophrenia *per se *is a risk factor for hypothermia [10]. This could also have contributed to hypothermia in our patient. Unlike our patient, who was on olanzapine for six years without any recent dose adjustment, previous case reports were associated with either initiation or dose adjustment of APD. Furthermore, there was no history of drug overdose in our patient. In this case, there were no risk factors for the development of hypothermia in the history, examination or investigations, other than the use of olanzapine and being a patient with schizophrenia.

## Conclusion

Hypothermia is a recognized complication of olanzapine, but this case report raises the possibility of hypothermia even in patients who are on stable doses of olanzapine for a long period of time.

## Consent

Written informed consent was obtained from the patient for publication of this case report and accompanying Figures 1 and 2. A specimen of the consent form is available for review by the Editor-in-Chief of this journal.

## Competing interests

The authors declare that they have no competing interests.

## Authors' contributions

RMLRR, HW, KTDPRJ, DMCG and DLBD made substantial contributions to the conception, design, acquisition of data and revisions. SLR, AC, DN and NPAKN contributed significantly to the acquisition of data and drafting the manuscript. All authors read and approved the final manuscript.
